# From the Patient’s Point of View

**Published:** 1909-04

**Authors:** George M. Gould

**Affiliations:** Ithaca, N. Y.


### BUFFALO MEDICAL JOURNAL.

Vol. LXIV. APRIL, 1909. No. 9

### ORIGINAL COMMUNICATIONS.

### From the Patient's Point of View

By GEORGE M. GOULD, M. D., Ithaca, N. Y.

THIS patient's history from the physician's standpoint was so instructive that I asked her to write it exactly as it appeared to her. When it was sent to me I found that my own records could add or change nothing, and as she is a woman of intellect and acumen, I have thought it best to let her account stand without change.

Ithaca, N. Y., February 7, 1909.
"Netherby," Cornell Heights.

My dear Dr. Gould: The accompanying is a full statement of the condition of my health from childhood to the time when I went to you last year at the age of 39 years.

I had been a child of good strong constitution, with a sound body, but of high strung nervous temperament, irregular in mood, full of wild spirits one day, depressed and melancholy without any cause, the next. My appetite was as capricious as my temper; sleep-walking and sleep-talking were of habitual nightly occurrence. The years between the ages of twelve and fifteen were spent in a boarding school. Continual headaches and severe pain in the face interrupted my classes, until finally all regular work was abandoned and it was an accepted fact that I should work one week and, alone in a dark room, rest completely the next. This meant giving up the use of my eyes, either for reading or writing, and even sewing.

Married at nineteen, the birth of my only child at twenty marked the breakdown of my physical strength, and during the next twenty years there was a gradual but constant deterioration of nerves and health, although no organic trouble ever developed and no disease was ever discovered to account for all the suffering. The nine months of pregnancy were marked by continual vomiting of unusual violence and the loss in weight of forty pounds. Nevertheless, I was able to nurse my child for eight months and she was fully and completely nourished.

The ten years following were made miserable by neuralgic pains in the face, under the right eye,—"tic douloureux" the doctors called it, and the habit of vomiting begun during pregnancy continued every morning. I was advised to have the suffering nerve cut, but as this was merely an experiment, I did not consent to have it done. The pain during those years became at times intolerable and the habit of taking small doses of morphine (1-16 of a grain hypodermatically) was commenced; during these years it was never permanently given up although the habit did not get beyond my control and the dread of it remained a wholesome restraint.

During my twenty-ninth year I edited a German book which involved three months of close work, mainly consisting of the reading of German type and the correction of proof-sheets. The result was an attack of pain so much more acute that it was followed by "nervous prostration" which kept me in bed for three months. The treatment of this illness consisted of 240 grains of bromide taken every two hours for twenty-four hours, with aconite and gelsemium on the alternating hours.

The result was that in six weeks I was taken to the Adirondacks reduced to "the lowest possible ebb of nerve-force," the effect aimed at by the bromide treatment. Memory and speech were impaired, feeling and sensibilities were dulled and the condition was one of continual "bromide drunkenness." Mine might have been taken for a case of erysipelas, because my face, head, hands and mouth were broken out with an eruption due to "bromide poisoning." But the neuralgic pain was gone! and in time I recovered, but to remain shattered nervously and so hardened to drugs that only enormous doses would ever have any future effect. From that date the hypodermic doses of morphine were increased to one grain and chloral for sleeplessness had to be taken in eighteen to twenty grain doses.

A trip to California and Europe was tried in order to take me from all social and household cares and work. The trip was unprofitable throughout because inability to take sufficient food to nourish me kept my vitality low, and my strength was being constantly sapped by headaches and vomiting.

In 1904 my daughter died and, soon after, a second attack of "nervous prostration" followed. This time there was no neuralgia in the face but a violent pain in the back of the head; this never completely left me until I put on glasses. The vomiting increased, and menstrual flowing continued uninterruptedly for eleven months. One month in bed again helped to reëstablish some degree of health and I began a régime consisting of two raw eggs and one pint of predigested milk a day. Such an insufficient diet would not have been possible had I not by medical advice taken whiskey in ever-increasing quantity,—the theory being that by taking alcohol I was saving my system "the necessity of manufacturing animal heat."

Two years later came a third attack of "nervous prostration" with all of the same symptoms as before, *i. e.*, violent pain in the head, nausea, uninterrupted menstrual flowing, sleeplessness, and the like. This time the nervous exhaustion was more complete and a trained nurse was needed for three months, her sole duty being to keep me from making any exertion, to feed me in minute quantities, and to give me morphine hypodermatically when the pain became intolerable.

The recovery was slower than on previous occasions and the nervous strain left was difficult to bear. An unexpected change in material circumstances made it necessary, January 1, 1908, for me to assume upon myself the whole burden of household work. Much to my surprise I was perfectly able to do it, and by giving up all reading, sewing, and literary work, and merely using my strength for manual labor, with no eye-work at all, I was not only able to do the work, but gradually improved both nervously and physically.

Imagine my disappointment when after three months of hopeful improvement I noticed that by slow degrees all of the old symptoms were returning, although no change had been made either in my work or in my manner of living. Sleeplessness returned, vomiting, the pain in the head and the continued menstrual flowing.

Then for the first time I had to notice that both my will-power and my mind were being affected. I could mark the mental deterioration by the difficulty I had in reading. The eyes could see, but the brain failed to be interested. Next it became wearisome for me to listen even to a short conversation, as my mind would wander from the speaker into blankness and a dazed, stupid vacancy. Memory for even ordinary daily duties failed me more and more often.

There must have been some kleptomania at this time, for on two occasions I found in my possession articles of small value, but which certainly had never belonged to me, and which I never could account for; I did remember deliberately taking one certain object which I *knew* was not mine, and which I did not in the least want or covet, but felt impelled to take because the temptation appeared strong and my will power had become numbed.

Lastly the memory of my own name and house left me suddenly while out in the street and I was unable to return home without assistance, because the locality of my house, the way back to it, all, had faded away leaving no trace in my memory.

The misery, terror, and hopeless horror of those weeks defy description. It had been easy to endure suffering, weakness, and the constant handicap of chronic ill-health. I had even been able to retain some hope against the hopelessness of a life-time of illness that had no name and could neither be cured nor helped. But to lose my mind and my will power was unendurable. My pride had been in my ability to endure physical suffering, and my firmness of purpose had served me valiantly for twenty years.

The tragedy of the future seemed to mean either insanity, or, in case I was driven again into the hands of the neurologist or specialist, into the morphine habit; for I well knew that even a small dose given to me in my enfeebled mental condition would inevitably be my absolute ruin. Then it was that I resolved to consult you, Dr. Gould, as a final hope, and if you had found that you could not help me, I was firmly resolved to commit suicide.

Before going to your office some necessary letters were written, my affairs were all left in perfect order, and an overdose of morphine lay in my desk awaiting the result of my interview with you.

You never had a patient come to you more determined not to become a mental wreck, or a morphine victim; never one whose life hung by a slenderer thread.

Up to this time I had been in the hands of ten different doctors, not one of whom had ever suggested that I might be suffering from the results of eyestrain. At the ages of 30 and 35 I had consulted two of the leading oculists of the country, as my eyes seemed for a short time to be affected by the "bromide cure" I had been through. After examining my eyes and testing my vision both oculists assured me that my "eyes were perfect." "The optic nerve is weak because your general health is poor. Go home, take care of yourself, build yourself up and your eyes will improve correspondingly,"—this was in substance their advice.

Your diagnosis of my case was that I had suffered all my life from severe eyestrain, due to a small amount of astigmatism and considerable accommodational strain.

Your prescription reads:

R. + S. 0.37 + C. 0.25 ax. 165°
L. + S. 0.37 + C. 0.25 ax. 180°

The relief to the intense nervous strain was instantaneous. From the first hour of wearing the glasses the tension was lifted and has never returned. Within two days the mental condition began to improve. Reading became easy, thinking and working were possible as of old. My memory returned as sharp and clear as ever, and never for an hour has there been any return of the horrible depression; kleptomania vanished; and also of course, all suicidal purpose. No morphine, drugs, or stimulants have been desired or taken since this time.

The improvement of a physical nature is even more marked, and more easily noted. The vomiting ceased *absolutely*. For the first time in twenty years food could be taken in sufficient quantity to nourish the body and supply strength and vitality.

Before the end of the first week the menstrual flowing that had been going on for five months ceased, and the past three months have proved that all irregularity has been completely corrected.

As for the pain in the head there has been no return of it at any time. I even note that in such an unimportant a matter as a nervous trembling of the hands, which had made writing and sewing difficult for many years, has entirely disappeared.

A gain in weight of eleven pounds during the first six weeks is proof positive of the completeness of my recovery.

Pray do not hesitate to make what use you may see fit of this long account of my condition. I should be very glad to have the pleasure of being in any way of service to you. My name and address are entirely at your disposal and I make absolutely no restrictions as to the manner in which you may deem it best to publish this.

Faithfully, yours always,
KATHERINE M. HEWETT.

One is in duty bound to add that there are large numbers of oculists and general physicians who have in the same way failed to cure thousands of patients afflicted with similar sufferings due to the same cause. Multitudes of patients are being passed through oculists' offices with the same diagnoses of "eyes are perfect," "too little astigmatism to correct," "it is all due to your general health,"—or they are outfitted with glasses which add insult to the organism's already-existing injury. The great "leaders" whom this patient consulted are still giving the same advice to thousands. The fashionable neurologists are still treating their patients in the same way, and the professional responsibility for morphinomania, drunkenness, bromidism, "neurasthenia," "hysteria," and the rest, continues and increases. Certainly several thousand persons are annually committing suicide to be relieved of the terror of insanity, and of hopeless, mysterious, "causeless" disease, invalidism, and tragedy, when eyestrain, and eyestrain alone is the easily demonstrable cause.

It is almost incredible that this patient should have escaped the gastrologist, the surgeon, or the gynecologist. This miracle was due to the sound judgment, even medical and scientific judgment, of the woman herself.

